# Inhibition of ferroptosis by *N*-oxide-based fluorescent probes *via* selective oxidation of ferrous ions†

**DOI:** 10.1039/d4sc07972h

**Published:** 2025-05-14

**Authors:** Kanta Kawai, Rie Haruki, Shunsuke Nozawa, Hideko Nagasawa, Tasuku Hirayama

**Affiliations:** a Laboratory of Pharmaceutical and Medicinal Chemistry, Gifu Pharmaceutical University 1-25-4, Daigaku-nishi Gifu 501-1196 Japan; b Institute of Materials Structure Science, High Energy Accelerator Research Organization 1-1 Oho Tsukuba Ibaraki 305-0801 Japan; c Precursory Research for Embryonic Science and Technology (PRESTO), Japan Science and Technology Agency Kawaguchi Saitama 332-0012 Japan hirayamat@gifu-pu.ac.jp

## Abstract

Ferroptosis, a form of iron-dependent programmed cell death, is linked to various diseases and physiological processes. Despite the availability of various types of ferroptosis inducers, its inhibitors are limited to iron chelators or radical scavengers. This study demonstrates that Fe(ii)-selective fluorescent probes developed in our lab could serve as ferroptosis inhibitors *via* selective oxidation of Fe(ii), which is required for lipid peroxidation. This finding opens the door to a new class of ferroptosis inhibitors with potential therapeutic applications.

## Abstract 2

Selective oxidation of ferrous ion by *N*-oxide inhibits ferroptosis in organelle-specific manner.

## Graphical abstract

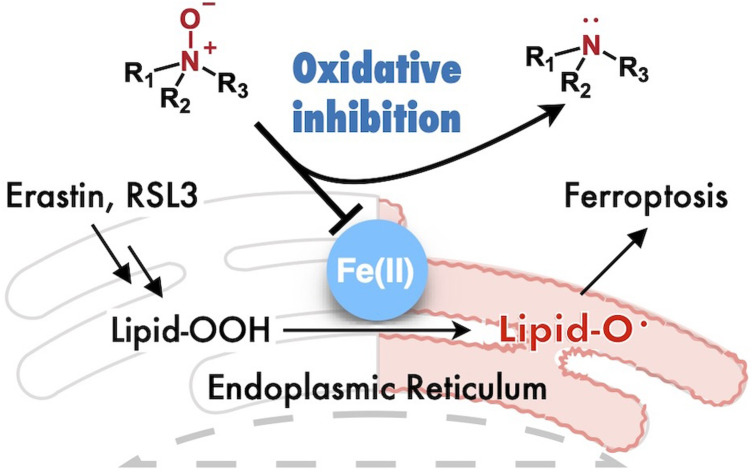

## Introduction

**Fig. 1:**
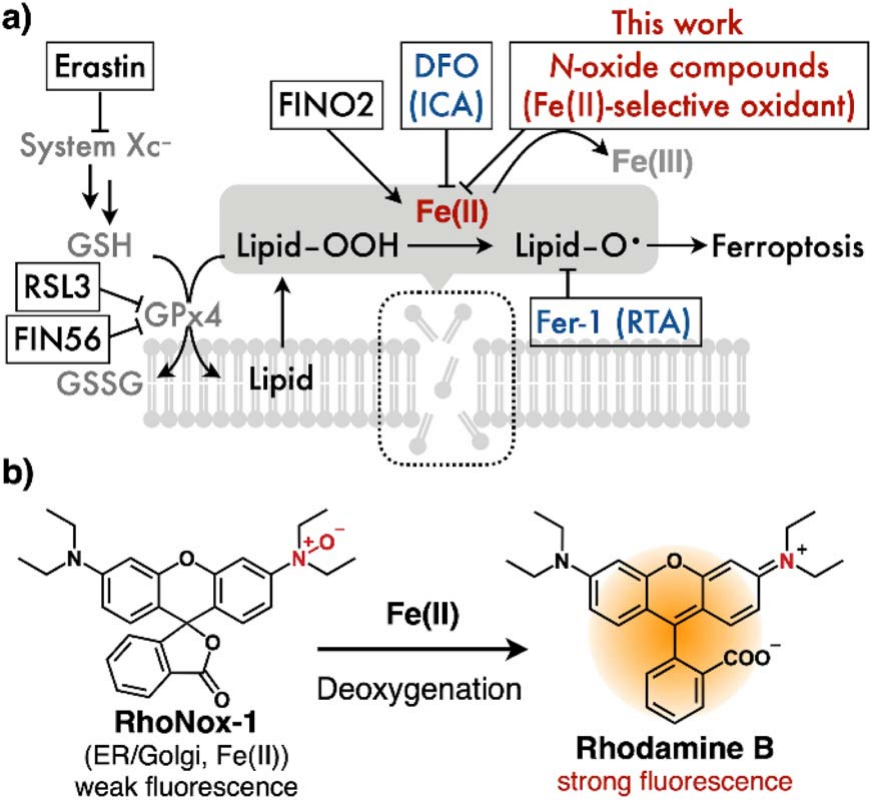
(a) Schematic representation of the ferroptosis pathway. GSH: glutathione, GSSG: gluotahione disulfide, GPx4: glutathione peroxidase 4, DFO (deferoxamine, ICA: iron-chelating agents), and Fer-1 (ferrostain-1, RTA: radical-trapping agents). Erastin and RSL-3 are ferroptosis inducers. (b) Structure and mechanism of RhoNox-1, a Fe(ii)-selective fluorescent probe based on *N*-oxide chemistry.

Ferroptosis, an iron-dependent form of programmed cell death, was first characterized by Stockwell *et al.* in 2012.^1^ This distinct cell death pathway is defined by the accumulation of lipid peroxides, which leads to plasma membrane disruption through Fenton-type reactions between ferrous ions (Fe(ii)) and phospholipid hydroperoxides (Fig. 1a). Accumulating evidence suggests that iron dysregulation and subsequent ferroptosis contribute to the pathogenesis of various disorders, including neurological diseases, ischemia–reperfusion injury, and glaucoma.^2,3^ Consequently, ferroptosis has emerged as a promising therapeutic target. The field of ferroptosis research was initiated with the discovery of erastin, which induces ferroptosis by inhibiting the System Xc^−^ transporter. Subsequently, additional ferroptosis inducers such as RSL3 (ref. 4) and FIN56 (ref. 5) were identified (Fig. 1a). Current ferroptosis inhibitors can be classified into two categories: iron-chelating agents (ICAs) and radical-trapping agents (RTAs) (Fig. 1a). Deferoxamine (DFO), a clinically approved ICA, was the first compound shown to inhibit erastin-induced cell death, leading to the term “ferroptosis.”^1,6^ Although antioxidants, including RTAs, have demonstrated efficacy in neurogenerative disease models, their clinical trials have yielded disappointing results.^7,8^ Moreover, these compounds often possess electron-rich lipophilic structures that can generate quinone-like metabolites, potentially causing hepatotoxicity and agranulocytosis.^9^ Although DFO exhibits neuroprotective properties,^10^ its clinical utility is limited by poor oral bioavailability due to rapid renal clearance. Second-generation ICAs, such as deferiprone (DFP) and deferasirox (DFX), were developed to address these limitations. However, their high binding stoichiometry requirements (3 : 1 for DFP : Fe and 2 : 1 for DFX : Fe) necessitate high doses, which can lead to adverse effects including granulocyte deficiency and renal failure.^11,12^ Furthermore, ICAs may inherently reduce iron availability, potentially causing anemia. These limitations underscore the urgent need for novel ferroptosis inhibitors with improved therapeutic profiles.

Our laboratory has developed a series of fluorescent probes for detecting labile Fe(ii) based on tertiary amine *N*-oxide (*N*-oxide) chemistry.^13–18^ These probes operate through an Fe(ii)-mediated deoxygenation mechanism that triggers fluorescence emission (Fig. 1b and Scheme S1†). The *N*-oxide-based detection system exhibits exceptional Fe(ii) selectivity and is highly compatible with live-cell imaging applications.

Through the strategic development of organelle-targeted probes, we have previously demonstrated elevated labile Fe(ii) concentrations in the endoplasmic reticulum (ER) and lysosomes during erastin-induced ferroptosis.^19^ More recently, we have expanded our *N*-oxide-based strategy to include H-FluNox^20^ for selective detection of labile heme and SiRhoNox-1 (ref. 21) (Scheme S1†), a far-red fluorescent probe specific to labile Fe(ii), enabling simultaneous monitoring of labile heme (LH) and labile Fe(ii) pools during ferroptotic process. Notably, Ikeda and colleagues reported that two of our commercially available Fe(ii)-selective fluorescent probes, Ac-MtFluNox and SiRhoNox-1, exhibited protective effects against ferroptosis in myocardial cells challenged with doxorubicin and ischemia/reperfusion injury.^22,23^ While initially attributed to Fe(ii) chelation, this mechanism requires revision as these *N*-oxide-based probes function as activity-based sensors without chelating moieties. Moreover, their anti-ferroptotic effects have been documented only in specific experimental models, and the underlying molecular mechanism remains to be elucidated. The present study aims to reveal the mechanistic basis of these probes' anti-ferroptotic activity from a chemical perspective and to comprehensively evaluate the ferroptosis-inhibitory potential of our *N*-oxide-based fluorescent probe library.

### XANES spectroscopy of the iron redox state upon reaction between Fe(ii) and *N*-oxide

**Fig. 2:**
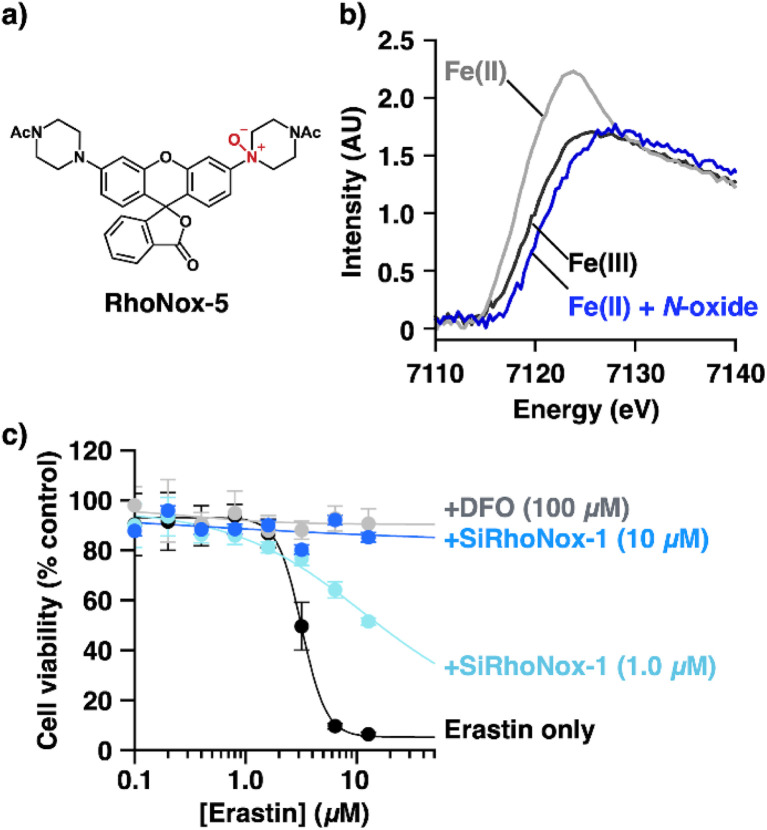
(a) Chemical structure of RhoNox-5. (b) X-ray Absorption Near Edge Structure (XANES) spectra of ferrous ammonium sulfate (FAS; Fe(ii), 4.0 mM) (gray line), a mixture of RhoNox-5 (4.0 mM) and FAS (4.0 mM) (blue line) after incubation for 30 min, and FeCl_3_ (4.0 mM) (black line) in water (20%DMF v/v). (c) Cell viability curves of HT1080 cells treated with erastin (12 h treatment) in the presence of SiRhoNox-1 (1.0 or 10 μM) or deferoxamine (DFO) (100 μM). Error bars indicate ±s.e.m (*n* = 4).

Our previous studies demonstrated that the *N*-oxide moiety in our probes undergoes deoxygenation upon reduction by Fe(ii).^13^ This reaction necessarily involves the oxidation of Fe(ii), presumably to Fe(iii) or a ferryl intermediate. To determine the oxidation state of iron following its reaction with *N*-oxide-based probes, we performed X-ray absorption near-edge structure (XANES) spectroscopic analysis. RhoNox-5 (Fig. 2a) was selected as the representative probe for this investigation due to its superior water solubility, which was essential for achieving the millimolar concentrations required for XANES measurements—substantially higher than those typically employed in UV-vis absorption and fluorescence spectroscopic analyses.^15^ XANES spectra were acquired for two experimental conditions: (1) Fe(ii), supplied as ferrous ammonium sulphate (FAS, 4 mM) in an aqueous solution containing 20% v/v DMF as co-solvent, and (2) the reaction mixture of RhoNox-5 (4 mM) and FAS (4 mM) under identical solvent conditions. Comparative analysis revealed a distinct shift of the absorption edge toward higher energies in the reaction mixture (Fig. 2b and S1†). When benchmarked against the XANES spectrum of ferric chloride, serving as an Fe(iii) reference standard (Fig. 2b and S1†), the data demonstrated the oxidation of Fe(ii) to Fe(iii) upon reaction with the *N*-oxide moiety.

### Inhibitory activity of *N*-oxide-based fluorescent probes for erastin-induced ferroptosis

**Fig. 3:**
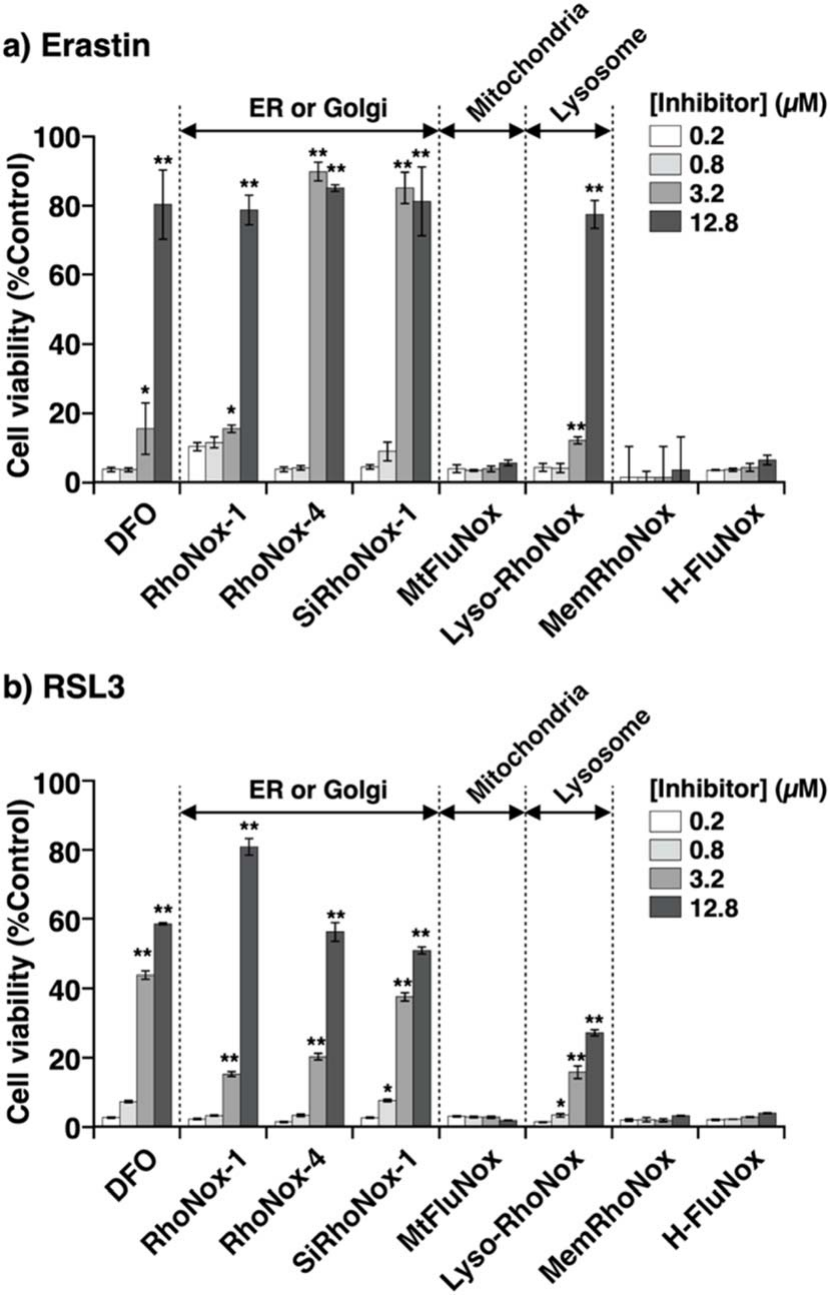
Inhibitory activity of the *N*-oxide probes toward (a) erastin- and (b) RSL3-induced ferroptosis. Fe(ii) fluorescent probes: RhoNox-1, RhoNox-4, SiRhoNox-1, MtFluNox, Lyso-RhoNox, and MemRhoNox. Labile heme fluorescent probe: H-FluNox. HT1080 cells were treated with the probes in the presence of erastin (10 μM, a) or RSL3 (10 μM, b) for 12 hours, and then cell viability was evaluated using CCK8. Statistical analysis was performed using Dunnett's test. *P < 0.01 and **P < 0.001. Each bar shows the mean ± s.e.m (*n* = 4).

We postulated that *N*-oxide chemistry could provide a novel therapeutic strategy against ferroptosis through the selective oxidation and subsequent depletion of Fe(ii), a crucial mediator of lipid radical generation. To test this hypothesis, we initially assessed the protective effects of SiRhoNox-1 against erastin-induced ferroptosis in HT1080 cells (Fig. 2c) because RhoNox-5 is impermeable to cell membranes due to its high hydrophilicity.^15^ In the absence of intervention, erastin exhibited an IC_50_ of 3.2 μM. Notably, SiRhoNox-1 demonstrated significant cytoprotective effects at concentrations as low as 1 μM, with complete cell rescue achieved at 10 μM (Fig. 2c). Furthermore, SiRhoNox-1 displayed superior inhibitory potency compared to the classical iron-chelating ferroptosis inhibitor, deferoxamine (DFO).^1^ Based on these promising results, we conducted a comprehensive evaluation of our *N*-oxide-based fluorescent probe library previously used in live-cell imaging studies, including RhoNox-1, RhoNox-4, SiRhoNox-1, MtFluNox, Lyso-RhoNox, MemRhoNox, and H-FluNox (Scheme S1†). For MtFluNox and H-FluNox, their acetylated derivatives (Ac-MtFluNox and Ac-H-FluNox) were employed to enhance cellular permeability. The assessment was performed under standardized conditions (10 μM erastin, 12-hour incubation) that reduced HT1080 cell viability to less than 10%. The protective efficacy of these probes exhibited marked structure-dependent variations. Quantitative analysis revealed distinct efficacy profiles among the tested probes. The assessment was performed under standardized conditions (10 μM erastin, 12-hour incubation) that reduced HT1080 cell viability to less than 10%. The protective efficacy of these probes exhibited marked structure-dependent variations. Quantitative analysis revealed distinct efficacy profiles among the tested probes. RhoNox-1 and Lyso-RhoNox demonstrated substantial ferroptosis inhibition with IC50 values of 12.8 μM, while RhoNox-4 and SiRhoNox-1 exhibited superior potency with IC_50_ values of approximately 3.5 μM (Fig. 3a). Notably, H-FluNox, Mem-RhoNox, and MtFluNox showed no protective effects across all tested concentrations. The effective inhibitors share a common rhodamine scaffold and display characteristic subcellular distribution patterns: RhoNox-1, RhoNox-4, and SiRhoNox-1 predominantly accumulate in the endoplasmic reticulum (ER) and/or Golgi apparatus, while Lyso-RhoNox specifically targets lysosomes. The enhanced efficacy of these organelle-specific probes correlates with previous observations that lipid peroxidation is initiated in the ER during early stages of ferroptosis.^24^ Intriguingly, the mitochondria-targeted probe MtFluNox showed no protective effect, in contrast to earlier findings in doxorubicin-induced ferroptosis of myocardial cells, where mitochondrial labile Fe(ii) accumulation was reported to play a crucial role.^23^ This discrepancy suggests that mitochondrial Fe(ii) elevation may be specific to doxorubicin-induced ferroptosis rather than a universal feature of ferroptotic cell death.

This organelle-specific response pattern aligns with our previous observation that mitochondrial labile Fe(ii) levels remain stable during erastin-induced ferroptosis in HT1080 cells.^19^ The inefficacy of MemRhoNox can be attributed to its membrane-anchored structure, where the *N*-oxide domain remains confined to the extracellular space,^17^ preventing access to the intracellular Fe(ii) pools that drive lipid peroxidation. The lack of protective effect observed with H-FluNox is consistent with our previous demonstration that labile heme does not directly participate in the execution of erastin-induced ferroptosis.^20^ This result further validates our understanding of the ferroptotic mechanism and the specificity of our probe-based intervention strategy.

Importantly, the deoxygenated analogues of these probes (Fig. S2a–c†) showed no significant protective effects (Fig. S2d†), confirming that the *N*-oxide moiety is the essential structural element for ferroptosis inhibition. This structure–activity relationship provides strong mechanistic support for our hypothesis that Fe(ii) oxidation, rather than alternative mechanisms, underlies the protective effects of these probes.

### Inhibitory activity against RSL-3-induced ferroptosis

To establish the broader applicability of *N*-oxide-based probes as ferroptosis inhibitors, we evaluated their protective effects against RSL-3-induced ferroptosis, which operates through glutathione peroxidase 4 (GPx4) inhibition.^4^ Experimental conditions were standardized to match those used in erastin studies (12-hour treatment), with the RSL-3 concentration (10 μM) selected based on dose–response studies that identified the point of maximal cell death (Fig. S3a†). In RSL-3-treated HT1080 cells, RhoNox-1, RhoNox-4, and SiRhoNox-1 demonstrated robust inhibitory activity, reducing cell death by more than 50%. Lyso-RhoNox exhibited moderate protection, achieving 30% inhibition at the highest tested concentration (12.8 μM) (Fig. 3b). Several critical control experiments validated the specificity of these effects: (1) the corresponding rhodamine and rhodol derivatives lacking the *N*-oxide moiety showed no protective effects (Fig. S3b†), and (2) the active *N*-oxide probes displayed no intrinsic cytotoxicity across the concentration range employed in inhibition studies (Fig. S4†). In addition, we employed FIN56 (ref. 5) and FINO2 (ref. 25) as ferroptosis inducers, which act as an indirect GPX4 degrader and an Fe(ii)-mediated radical generator, respectively. The effective probes, RhoNox-1, RhoNox-4, and SiRhoNox-1 exhibited comparable inhibitory activity (Fig. S6†). The consistent inhibitory activity of these probes against ferroptosis induced by erastin, RSL-3, FIN56, and FINO2 suggests that their inhibitory effects are independent of the specific ferroptosis pathway and cover four major types of ferroptosis (Fig. 1a).^26^ Furthermore, these results reinforce our previous conclusion that both the *N*-oxide moiety and appropriate subcellular targeting—specifically to the ER/Golgi apparatus or lysosomes—are essential structural requirements for effective ferroptosis inhibition.

**Fig. 4:**
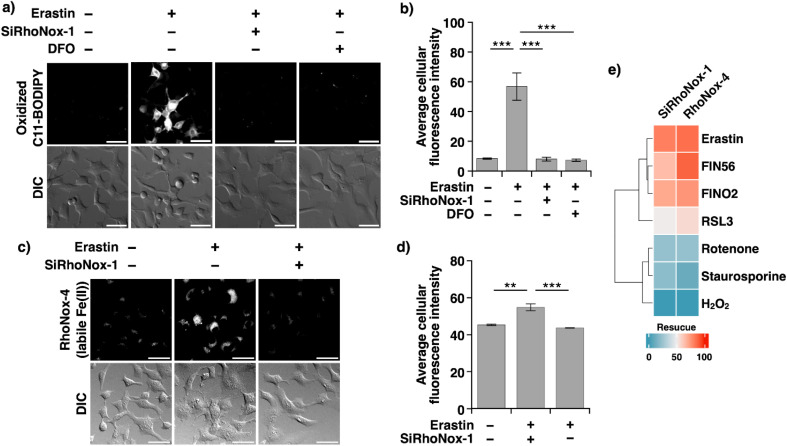
(a) Confocal fluorescence microscopy images (top) of C11 BODIPY and differential interference contrast (DIC) images (bottom) of HT1080 cells pretreated with erastin (10 μM) in the presence of SiRhoNox-1 (5.0 μM) or DFO (100 μM) for 6 h before washing and staining with C11-BODIPY (1.0 μM) in HBSS for 1.0 h, followed by washing and imaging. Scale bars indicate 25 μm. (b) Mean fluorescence intensities were quantified for the images obtained in the experiment (a). Each bar shows the mean ± s.e.m (*n* = 5). Statistical analysis was performed using Tukey's test. ***P < 0.001. (c) Confocal fluorescence microscopic images (top) of RhoNox-4 and DIC images (bottom) of HT1080 cells pretreated with erastin (10 μM) in the presence of SiRhoNox-1 (10 μM) for 6 h before washing and staining with RhoNox-4 in HBSS for 1.0 h, followed by washing and imaging. Scale bars indicate 25 μm. (d) Mean fluorescence intensities were quantified for the images obtained in the experiment (c). Each bar shows the mean ± s.e.m (*n* = 3). Statistical analysis was performed using Tukey's test. **P < 0.01 and ***P < 0.001. (e) Effect of SiRhoNox-1 and RhoNox-4 (12.8 μM) on the cell viability of HT1080 cells treated with different lethal compounds under lethal conditions.

To directly assess the impact of *N*-oxide-based probes on the hallmark feature of ferroptosis—Fe(ii)-dependent lipid peroxidation—we employed C11-BODIPY-based fluorescence microscopy. SiRhoNox-1 was selected for these studies due to its far-red emission profile, which prevents spectral overlap with C11-BODIPY. HT1080 cells were exposed to erastin (10 μM) with or without SiRhoNox-1 (5.0 μM) for 6.0 hours, using DFO as a benchmark inhibitor. Fluorescence imaging analysis revealed that the erastin-induced increase in lipid peroxidation was effectively suppressed by SiRhoNox-1, with inhibition levels comparable to those achieved by DFO (Fig. 4a and b). Similar suppression patterns were observed in RSL-3-induced ferroptosis (Fig. S7†). Then, we explored intracellular labile Fe(ii) levels during erastin-induced ferroptosis using SiRhoNox-1 as an inhibitor and RhoNox-4 as a fluorescent probe for monitoring labile Fe(ii) in living cells. The fluorescence intensity increased upon the treatment with erastin (10 μM, 6 h), indicating upregulation of labile Fe(ii) levels, while it remained unchanged when SiRhoNox-1 was co-treated with erastin (Fig. 4c and d). These findings provide direct evidence that SiRhoNox-1 prevents ferroptotic cell death by suppressing Fe(ii)-dependent Fenton-like radical reactions through the selective oxidative depletion of the catalytic Fe(ii) pool.

To establish the specificity of our *N*-oxide-based probes for ferroptosis, we evaluated their effects on alternative cell death modalities. HT1080 cells were treated with established inducers of necrosis (hydrogen peroxide, 5.0 μM, 12 hours) or apoptosis (staurosporine, 1.0 μM, or rotenone, 64 μM, 24 hours) in the presence or absence of RhoNox-4 or SiRhoNox-1. Notably, neither probe demonstrated protective effects against apoptotic or necrotic cell death, confirming their selective inhibition of the ferroptotic pathway (Fig. 4d).

## Conclusions

In this study, we have established that *N*-oxide-based probes can effectively inhibit ferroptosis through selective oxidation and subsequent depletion of Fe(ii). This mechanism represents a novel approach to ferroptosis inhibition, distinct from previously reported strategies.^27^ The versatility of *N*-oxide chemistry suggests potential for broader application across diverse tertiary amine compounds. Notably, the existence of FINO-2, a ferroptosis inducer containing an endoperoxide motif^25^ (also employed in other Fe(ii)-selective fluorescent probes^28,29^) suggests an intriguing possibility that endoperoxide- and *N*-oxide-based compounds could serve as complementary tools for modulating cellular labile Fe(ii) homeostasis. Our systematic evaluation of organelle-targeted *N*-oxide probes revealed that compounds localizing to the ER/Golgi apparatus and lysosomes effectively inhibit both erastin- and RSL-3-induced ferroptosis. This consistent protection across different ferroptosis inducers suggests that Fe(ii)-catalysed Fenton-type reactions occur at these specific organelles as a common downstream event in the ferroptotic cascade. This finding aligns with previous reports identifying the ER as a primary site of lipid peroxidation and complements earlier studies using organelle-targeted RTAs^30^ and lipid peroxide inhibitors.^24^ The unique mechanism of our *N*-oxide-based probes—direct intervention in Fe(ii)-dependent processes—provides a powerful tool for investigating ferroptotic cell death. This approach offers distinct advantages for elucidating both the cellular sources of catalytic Fe(ii) and the specific subcellular sites where lipid peroxidation is initiated, potentially leading to new therapeutic strategies for ferroptosis-related diseases.

## Author contributions

T. H. designed the study, the main concept, and the proof outline. K. K., R. H., S. N., and T. H. collected the data. K. K., R. H., S. N., H. N, and T. H. aided in interpreting the results and worked on the manuscript. T. H. supervised the project. K. K. wrote the manuscript with support from T. H. All authors discussed the results and commented on the manuscript.

## Conflicts of interest

There are no conflicts of interest to declare.

## Supplementary Material

SC-OLF-D4SC07972H-s001

## Data Availability

The data supporting this article have been included as part of the ESI.†
