# Supplementary material for: Inhibition of ferroptosis by N-oxide-based fluorescent probes via selective oxidation of ferrous ions
Source: Chem Sci. 2025 May 14;16(25):11240–5. doi: 10.1039/d4sc07972h (PMC12091573; doi:10.1039/d4sc07972h)
Supplement: SC-016-D4SC07972H-s001 [file SC-016-D4SC07972H-s001.pdf]

Supplementary Information (SI) for Chemical Science.
This journal is © The Royal Society of Chemistry 2025

Supporting information for

Inhibition of ferroptosis by *N*-oxide-based fluorescent probes via selective oxidation of ferrous ion

Kanta Kawai,[a] Rie Haruki,[b] Shunsuke Nozawa,[b] Hideko Nagasawa,[a] Tasuku Hirayama*[a,c]

[a]Laboratory of Pharmaceutical and Medicinal Chemistry, Gifu Pharmaceutical University, 1-25-4, Daigaku-nishi, Gifu-shi, Gifu, 501-1196, Japan
[b]Institute of Materials Structure Science, High Energy Accelerator Research Organization, 1-1 Oho, Tsukuba, Ibaraki, 305-0801, JAPAN
[c]Precursory Research for Embryonic Science and Technology (PRESTO), Japan Science and Technology Agency, Kawaguchi, Saitama, 332-0012 Japan
e-mail: hirayamat@gifu-pu.ac.jp

Contents

Supplementary figures and legends

Scheme S1. Chemical structures of *N*-oxide-based fluorescent probes
Figure S1. Cell viability assay of the parent dyes toward erastin-induced ferroptosis
Figure S2. Cell viability assay of RSL-3-induced ferroptosis and the effect of parent dyes
Figure S3. Cytotoxicity assay of the probes

Materials and methods
General
1. X-ray absorption near edge structure spectrum (XANES)
2. Cell culture
3. Cell viability assay
4. Confocal fluorescence microscopy experiments

Supplementary Figures

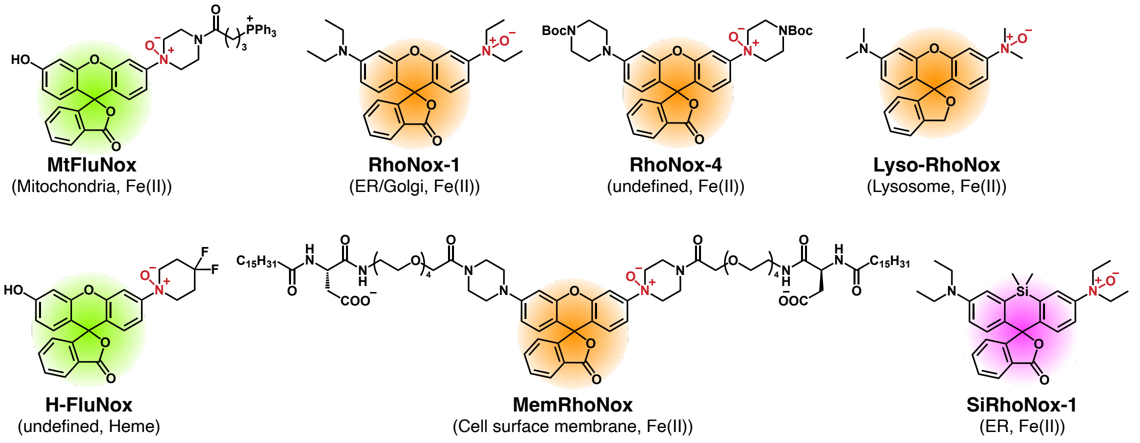

Scheme S1. Chemical structures, targeted organelles, and fluorescent colors of *N*-oxide-based fluorescent probes.

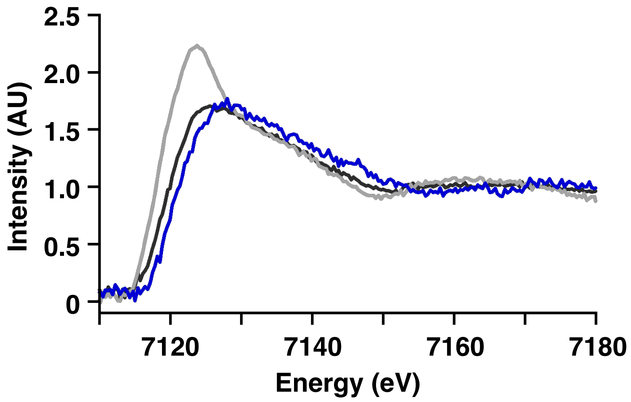

Figure S1. A Wide-range X-ray Absorption Near Edge Structure (XANES) spectra of ferrous ammonium sulfate (FAS; Fe(II), 4.0 mM) (gray line), a mixture of RhoNox-5 (4.0 mM) and FAS (4.0 mM) (blue line) after incubation for 30 min, and $FeCl_3$ (4.0 mM) (black line) in water (20%DMF v/v).

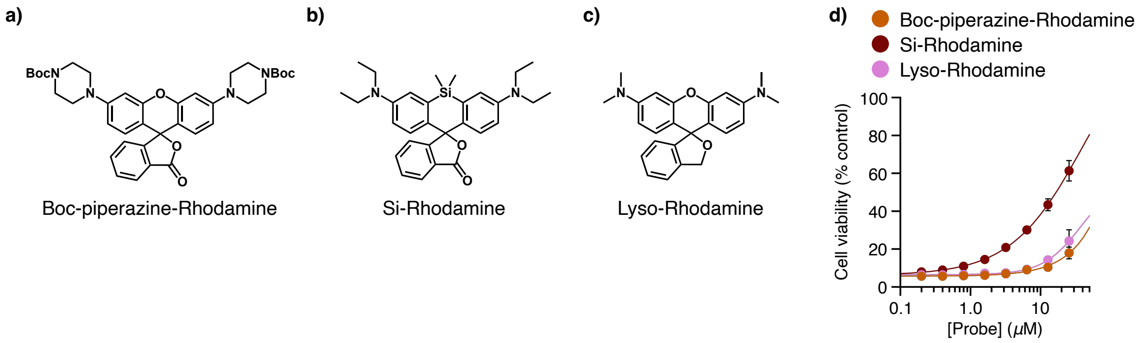

Figure S2. (a–c) Structures of the parent fluorophores of RhoNox-4, SiRhoNox-1, and Lyso-RhoNox. (d) Cell viability of HT1080 cells treated with erastin (10 μM) in the presence of the each deoxygenated compound for 12 h. Cell viability was measured by CCK8. Error bars indicate ±s.e.m (n = 4).

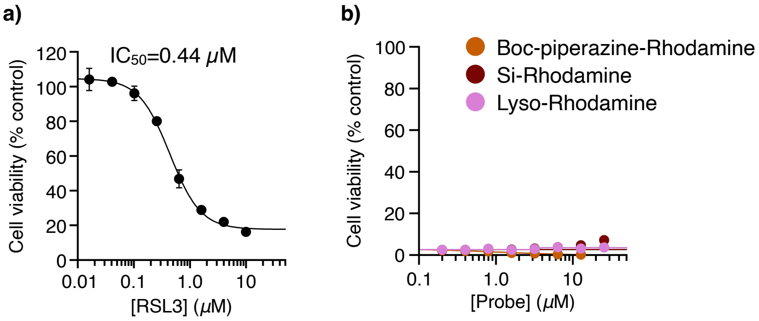

Figure S3. Cell viability curves of HT1080 cells treated with (a) RSL-3. (b) Cell viability of HT1080 cells treated with erastin (10 μM) in the presence of each deoxygenated compound as listed in Figure S2a–c for 12 h. Cell viability was measured by CCK8. Error bars indicate ±s.e.m (n = 4).

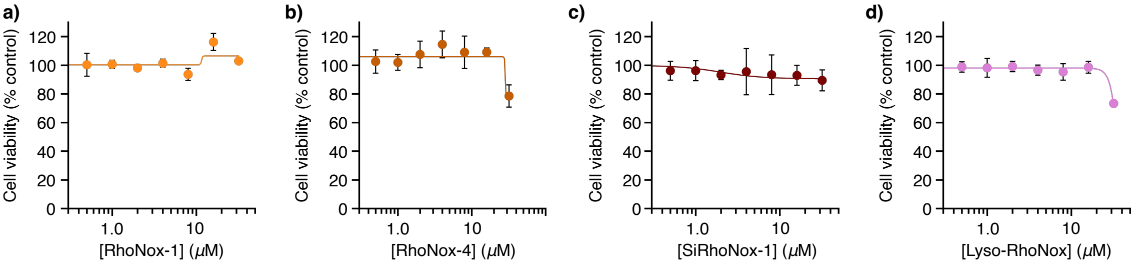

Figure S4. (a) Cell viability curve of HT1080 cells treated with RSL3 for 12 h. (b) Cell viability curves of HT1080 treated with RSL3 (10 μM) with the parent fluorophores shown in Figure S1a-c in for 12 h. Cell viability was measured by CCK8. Error bars indicate ±s.e.m (n = 4).

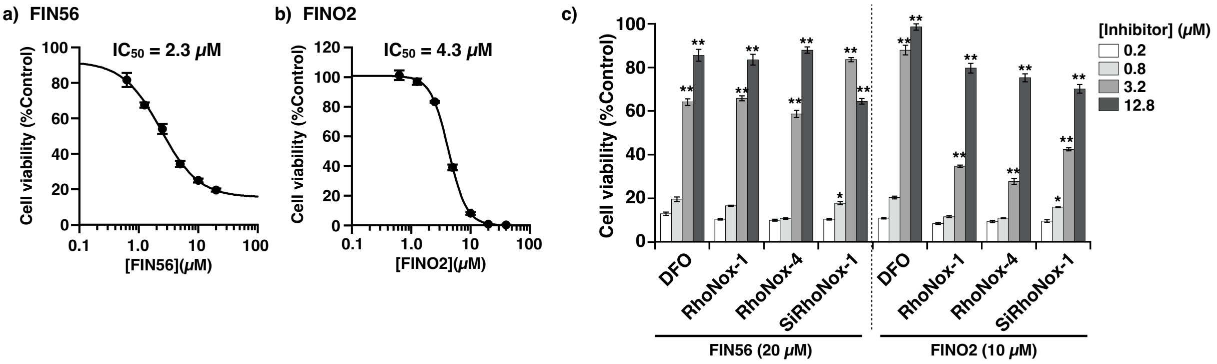

Figure S5. (a) Cell viability curve of HT1080 cells treated with FIN56 for 24 h (b) Cell viability curve of HT1080 cells treated with FINO2 for 2 h. (c) Inhibitory activity of the N-oxide probes toward (a) FIN56- and (b) FINO2-induced ferroptosis. HT1080 cells were treated with the probes in the presence of FIN56 (20 μM, a) for 24 hours or FINO2 (10 μM, right) for 12 hours, and then cell viability was evaluated by CCK8. Statistical analysis was performed using Dunnett's test. *$P < 0.01$, **$P < 0.001$. Each bar shows the mean ± s.e.m (n = 4).

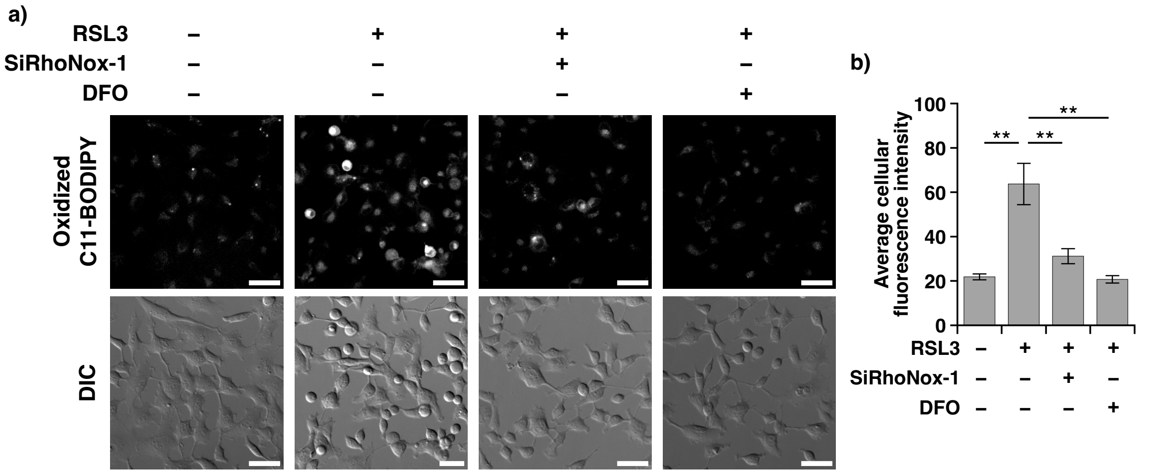

Figure S6. (a) Confocal fluorescence microscopic images (top) of C11 BODIPY and differential interference contrast (DIC) images (bottom) of HT1080 cells pretreated with RSL-3 (500 nM) in the presence of SiRhoNox-1 (5.0 μM) or DFO (100 μM) for 2 h before washing and staining with C11-BODIPY (1.0 μM) in HBSS for 1.0 h, followed by washing and imaging. Scale bars indicate 25 μm. (b) Mean fluorescence intensities were quantified for the images obtained in experiment (a). Each bar shows the mean ±s.e.m (n = 5). Statistical analysis was performed using Tukey's test. ***$P < 0.001$.

General

General chemicals used in this study were purchased from Fuji-film Wako Pure Chemical Co., Tokyo Chemical Industry Co., Kanto Chemical Co. Inc., and Aldrich Chemical Co., and were used without purification unless otherwise noted. The *N*-oxide-based fluorescent probes, including RhoNox-1,[1] Lyso-RhoNox,[2] RhoNox-4,[3] RhoNox-5,[3] SiRhoNox-1,[4] Mem-RhoNox,[5] MtFluNox,[6] and H-FluNox[7] were synthesized and used as previously reported in our lab. The synthetic schemes of these probes are described below.

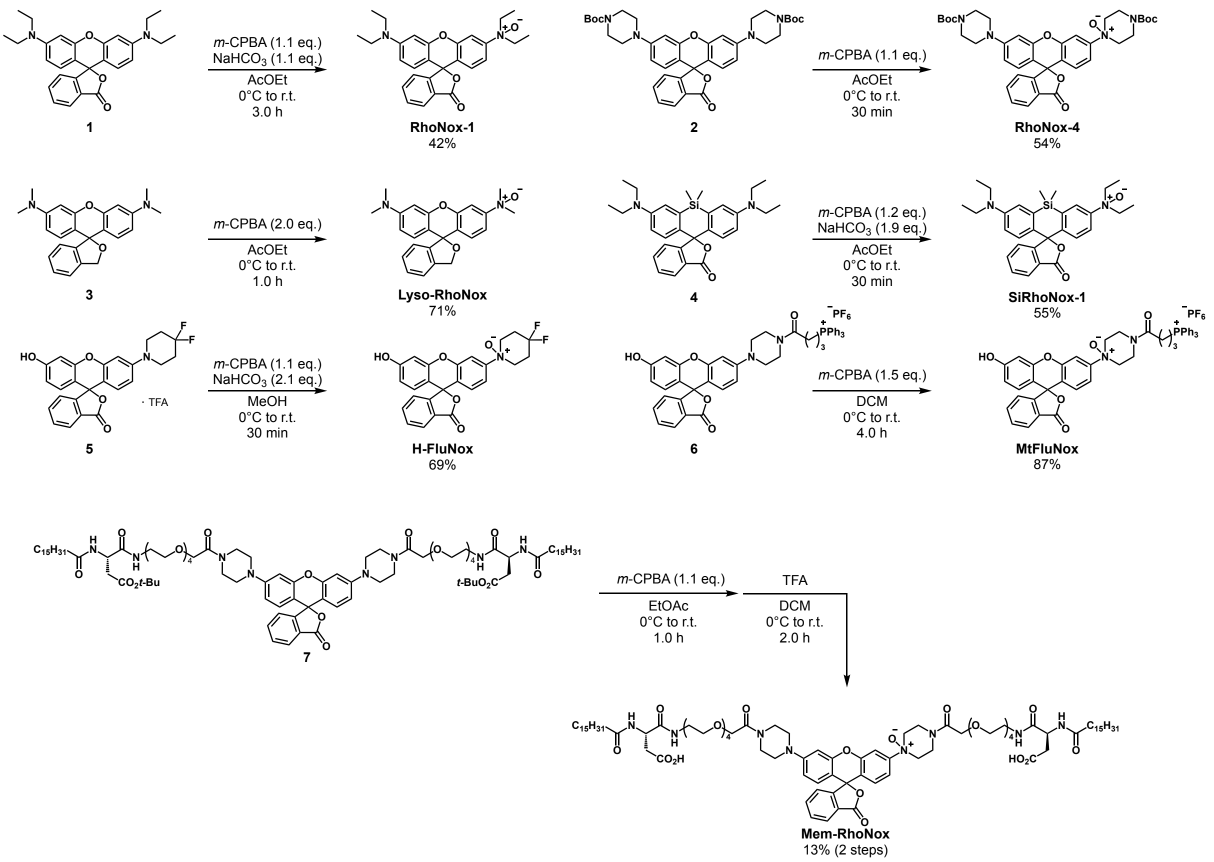

1. Synthesis of the *N*-oxide-based probes

RhoNox-1[1]

To a mixture of compound **1** (100 mg, 0.21 mmol) and sodium bicarbonate (19 mg, 0.23 mmol) in ethyl acetate (10 mL) was slowly added *m*-chloroperbenzoic acid (*m*-CPBA) (40 mg, 0.23 mmol) at 0 °C. The mixture was warmed to room temperature and stirred for 3 h. Insoluble materials were removed by filtration with a pad of celite, and then the filtrate was evaporated. The residue was purified with silica gel column chromatography ($CHCl_3$: MeOH = 10: 1) to afford RhoNox-1 as pale purple solid (40 mg, 42%). ^{1}H NMR (500 MHz, $CDCl_3$) δ : 1.18–1.13 (m, 12H), 3.36 (q, 4H, *J* = 6.9 Hz), 3.70 (brs, 4H), 6.38 (dd, 1H, *J* = 9.1, 2.3 Hz), 6.47 (d, 1H, *J* = 2.3 Hz), 6.58 (d, 1H, *J* = 8.6 Hz), 6.84 (d, 1H, *J* = 8.6 Hz), 7.21 (d, 1H, *J* = 7.4 Hz), 7.26 (br, 1H), 7.65 (t, 1H, *J* = 7.5 Hz), 7.69 (t, 1H, *J* = 7.4 Hz), 7.97 (d, 1H, *J* = 2.3 Hz), 8.04 (d, 1H, *J* = 7.5 Hz)

^{13}C NMR (125 MHz, $CDCl_3$) δ: 169.4, 152.8, 152.7, 149.9, 135.2, 129.9, 129.0, 128.9, 127.0, 125.2, 124.1, 120.4, 115.8, 120.4, 115.8, 112.6, 108.9, 104.5, 102.7, 97.7, 83.3, 67.1, 44.6, 12.6, 8.4.

HRMS (ESI+): *m/z* calculated for $C_{28}H_{31}N_2O_4^+$: 459.2278, found 459.2264.

RhoNox-4[2]

To a solution of compound **2** (114 mg, 0.17 mmol) in EtOAc (5 mL) was added *m*-CPBA (46 mg, 0.19 mmol) at 0 °C. After stirring at room temperature for 30 min, the reaction solvent was removed by evaporation. The residue was purified by silica gel column chromatography ($CHCl_3$: MeOH = 100 : 1 to 30 : 1) to afford RhoNox-4 as white powder (63 mg, 54%).

^{1}H-NMR (CD_3OD, 500 MHz) δ: 8.12 (d, *J* = 2.3 Hz, 1H), 8.04 (d, *J* = 7.4 Hz, 1H), 7.82–7.74 (m, 1H), 7.74–7.68 (m, *J* = 7.4 Hz, 1H), 7.65 (dd, *J* = 8.9, 2.6 Hz, 1H), 7.18 (d, *J* = 7.4 Hz, 1H), 6.97 (d, *J* = 9.2 Hz, 1H), 6.86 (d, *J* = 2.3 Hz, 1H), 6.76 (dd, *J* = 9.2, 2.3 Hz, 1H), 6.65 (d, *J* = 8.6 Hz, 1H), 4.27–4.08 (m, 2H), 4.08–3.94 (m, 2H), 3.94–3.67 (m, 2H), 3.67–3.44 (m, 4H), 3.24 (t, *J* = 5.2 Hz, 6H), 1.49 (s, 9H), 1.48 (s, 9H).

^{13}C-NMR (CD_3OD, 125 MHz) δ: 170.9, 156.8, 156.3, 155.6, 154.6, 154.1, 153.4, 153.1, 136.8, 131.5, 130.6, 129.7, 127.6, 126.1, 125.1, 122.0, 116.7, 114.0, 111.3, 109.6, 103.1, 83.6, 82.1, 81.4, 67.9, 45.0 (br), 43.9 (br), 40.4 (br), 39.2 (br), 28.7, 28.6.

HRMS (ESI+): *m/z* calculated for $[C_{38}H_{44}N_4NaO_8]^+$: 707.3051, found: 707.3028.

HM-RhoNox-M[3]

To a solution of compound **3** (90 mg, 0.24 mmol) in EtOAc (18 mL) was slowly added *m*-CPBA (83 mg, 0.48 mmol) at 0 °C. The mixture was warmed to room temperature and stirred for 1 h. The reaction mixture was evaporated, and then the residue was purified by alumina column chromatography ($CHCl_3$: MeOH= 50 : 1 to 20 : 1) to afford HMRhoNox-M as colorless powder (64 mg, 71%). ^{1}H-NMR (500 MHz, $CDCl_3$) δ : 7.93 (d, *J* = 2.3 Hz, 1H), 7.45 (dd, *J* = 8.6 Hz, 2.3 Hz, 1H), 7.39–7.38 (m, 2H), 7.29–7.26 (m, 1H), 7.05 (d, *J* = 8.6 Hz, 1H), 6.91 (d, *J* = 7.4 Hz, 1H), 6.81 (d, *J* = 8.6 Hz, 1H), 6.48–6.46 (m, 2H), 5.30 (dd, *J* = 20.0 Hz, 12.6 Hz, 2H), 3.58 (m, 6H), 2.98 (s, 6H)

^{13}C-NMR (125 MHz, $CDCl_3$) δ: 154.5, 151.5, 151.3, 150.9, 144.5, 139.1, 129.9, 129.2, 128.4, 128.2, 128.2, 125.7, 123.8, 120.7, 114.2, 111.7, 109.2, 109.0, 98.5, 83.3, 72.1, 63.2, 63.1, 40.3.

HRMS (ESI+): *m/z* calculated for $C_{24}H_{25}N_2O_3^+$: 389.1860, found 389.1871.

SiRhoNox-1[4]

To a mixture of compound **4** (300 mg, 0.6 mmol) and $NaHCO_3$ (104 mg, 1.2 mmol) in EtOAc (15 mL, 2.0 eq.) was slowly added *m*-CPBA (182 mg, 0.74 mmol, 1.2 eq.) at 0 °C. The mixture was warmed to room temperature, and then stirred for 30 min. Insoluble materials were removed by filtration with a pad of celite, and then the filtrate was evaporated. The residue was purified with silica gel chromatography ($CHCl_3$: MeOH = 15: 1) to give SiRhoNox-1 as an orange solid (172 mg, 55%).

^{1}H NMR (400 MHz, $CDCl_3$) δ: 8.31 (d, *J* = 2.4 Hz, 1H), 8.01 (d, *J* = 7.7 Hz, 1H), 7.72 (t, *J* = 7.0 Hz, 1H), 7.61 (t, *J* = 7.5 Hz, 1H), 7.49–7.36 (m, 2H), 7.01 (d, *J* = 9.2 Hz, 1H), 6.94 (d, *J* = 2.9 Hz, 1H), 6.83 (d, *J* = 9.2 Hz, 1H), 6.51 (dd, *J* = 9.2, 2.9 Hz, 1H), 3.78-3.54 (m, 4H), 3.37 (q, *J* = 7.1 Hz, 4H), 1.21–1.02 (m, 12H), 0.70 (s, 3H), 0,67 (s, 3H); ^{13}C NMR (100 MHz, $CDCl_3$) δ: 169.8, 152.5, 148.0, 146.5, 145.2, 138.4, 136.5,

133.5, 128.9, 128.1, 127.3, 126.6, 126.5, 125.8, 124.5, 121.7, 115.6, 112.1, 90.4, 66.5, 66.4, 43.9, 12.1, 8.0, −2.1.

HRMS (ESI+): *m/z* calculated for $C_{30}H_{37}N_2O_3Si^+$: 501.2568, found 501.2571

Mem-RhoNox[5]

To a solution of **7** (84 mg, 0.048 mmol) in EtOAc (9 mL) was slowly added *m*-CPBA (9.1 mg, 0.053 mmol) at 0 °C. The mixture was warmed to room temperature and stirred for 1 h. The reaction mixture was then cooled to 0 °C, and an additional portion of *m*-CPBA (3.3 mg, 0.019 mmol) was added. The mixture was warmed to room temperature and stirred for 0.5 h. After evaporation of the solvent, the residue was purified by silica gel column chromatography ($CHCl_3$: MeOH= 20 : 1 to 5 :1 to 3 : 1) to afford **7** as a pale purple solid (23 mg, 22%).

Then, to a solution of the obtained compound (7.3 mg, 0.0041 mmol) in CH_2Cl_2 (1 mL) was added TFA (1 mL) dropwise at 0 °C. After stirring at room temperature for 2 h, the mixture was evaporated, and then the residue was azeotroped with toluene. The dark red residue was purified by diol silica gel column chromatography ($CHCl_3$: MeOH = 100 : 1 to 50 : 1) to afford Mem-RhoNox as a pale pink solid (4.1 mg, 60%).

^{1}H-NMR (CD_3OD, 500 MHz) δ: 7.98–7.96 (m, 2H), 7.82–7.71 (m, 1H), 7.70 (t, *J* = 7.4 Hz, 1H), 7.65 (t, *J* = 7.2 Hz, 1H), 7.59–7.57 (m, 1H), 7.12 (d, *J* = 8.0 Hz, 1H), 6.99 (d, *J* = 8.6 Hz, 1H), 6.74–6.72 (m, 1H), 6.69 (dd, *J* = 8.6 Hz, 2.0 Hz, 1H), 6.58 (d, *J* = 8.6 Hz, 1H), 4.60–4.59 (m, 3H), 4.34 (d, *J* = 13.7 Hz, 1H), 4.20–4.14 (m, 6H), 4.00–3.98 (m, 1H), 3.61–3.37 (m, 39H), 3.25–3.20 (m, 4H), 2.81–2.75 (m, 2H), 2.71–2.68 (m, 2H), 2.57–2.53 (m, 2H), 2.12–2.11 (m, 4H), 1.48–1.47 (m, 4H), 1.18–1.16 (m, 48H), 0.79 (t, *J* = 6.9 Hz, 6H).

^{13}C-NMR (CD_3OD, 125 MHz) δ: 176.2, 173.9, 173.2, 170.8, 170.3, 170.2, 154.4, 153.9, 153.4, 153.3, 136.9, 131.7, 131.3, 129.8, 127.6, 126.2, 125.1, 123.1, 116.6, 116.5, 114.0, 111.4, 109.5, 103.0, 83.4, 71.7, 71.6, 71.52, 71.45, 71.41, 71.3, 71.2, 71.1, 70.9, 70.5, 70.4, 51.3, 45.7, 42.6, 41.2, 40.5, 37.9, 37.1, 37.0, 36.9, 33.1, 30.8, 30.6, 30.5, 30.4, 30.3, 26.9, 23.7, 14.5.

HRMS (ESI−): *m/z* calculated for $[C_{88}H_{135}N_8O_{22}]^-$: 1655.9696, found: 1655.9670.

MtFluNox[6]

Compound **6** (340 mg, 0.419 mmol) was dissolved in H_2O (100 mL), and a solution of KPF_6 (773 mg, 4.19 mmol) in H_2O (15 mL) was added to the solution. After stirring for 30 min, the mixture was filtered to obtain the corresponding PF_6^- salt (356 mg, 98%). Then, the PF_6^- salt (90 mg, 0.10 mmol) was dissolved in CH_2Cl_2 (10 mL). After stirring at 0 °C for 10 min, *m*-CPBA (26 mg, 0.15 mmol) was added. After stirring at room temperature for 4 h, the reaction mixture was concentrated, and the residue was purified by silica gel column chromatography ($CHCl_3$: MeOH= 10 :1) to yield **MtFluNox** (80 mg, 87%).

^{1}H NMR ($CDCl_3$, 500 MHz) δ: 8.10 (s, 1H), 8.01 (d, *J* = 7.4 Hz, 1H), 7.89–7.70 (m, 17H), 7.64 (d, *J* = 8.6 Hz, 1H), 7.20 (d, *J* = 7.4 Hz, 1H), 6.97 (d, *J* = 8.6 Hz, 1H), 6.73 (d, *J* = 2.3 Hz, 1H), 6.62 (d, *J* = 8.6 Hz, 1H), 6.57 (dd, *J* = 8.6, 2.3 Hz, 1H), 4.64 (d, *J* = 13.7 Hz, 1H), 4.08–3.95 (m, 4H), 3.65 (t, *J* = 9.9 Hz, 1H), 3.45–3.37 (m, 2H), 3.30–3.25 (m, 2H), 2.84–2.66 (m, 2H), 2.00–1.94 (m, 2H).

^{13}C NMR (CD_3OD, 125 MHz) δ: 171.8, 171.2, 161.7, 156.5, 154.1, 153.5, 153.0, 136.9, 136.3 (J_{PC} = 2.4 Hz), 134.8 (J_{PC} = 9.6 Hz), 131.6 (J_{PC} = 13.2 Hz), 131.5, 130.6, 130.2, 127.5, 126.1, 125.1, 122.2, 120.2, 119.5, 116.7, 114.3, 111.3, 110.6, 103.7, 83.6, 67.9 (J_{PC} = 37.2 Hz), 41.3, 37.8, 33.2 (J_{PC} = 16.0 Hz), 22.3 (J_{PC} = 52.8 Hz), 19.3.
HRMS (ESI+): *m/z* calculated for $C_{46}H_{40}N_2O_6P^+$: 747.2619, found 747.2611.

H-FluNox[7]
A suspension of compound **5** (232 mg, 0.423 mmol) and $NaHCO_3$ (72.7 mg, 0.866 mmol) in MeOH (10 mL) was heated at 40 °C until the material was dissolved. *m*-CPBA (78.2 mg, 0.453 mmol) was added to the mixture at 0 °C. After stirring at room temperature for 30 min, the mixture was evaporated, and the residue was purified by silica gel column chromatography ($CHCl_3$: MeOH = 1 : 0 to 30 : 1 to 10 : 1) to afford H-FluNox as a white solid (141 mg, 69%).
^{1}H NMR (500 MHz, DMSO-d6): δ 10.69 (s, 1H), 8.38 (d, *J* = 2.3 Hz, 1H), 8.05 (dd, *J* = 7.3, 1.4 Hz, 1H), 7.89 (dd, *J* = 8.7, 2.3 Hz, 1H), 7.81 (td, *J* = 7.3, 1.4 Hz, 1H), 7.76 (td, *J* = 7.3, 0.9 Hz, 1H), 7.34 (d, *J* = 7.3 Hz, 1H), 6.90 (d, *J* = 8.7 Hz, 1H), 6.78 (d, *J* = 1.8 Hz, 1H), 6.62 (d, *J* = 8.7 Hz, 1H), 6.59 (dd, *J* = 8.7, 1.8 Hz, 1H), 4.14 (q, *J* = 5.5 Hz, 1H, MeOH), 4.07 (t, *J* = 11.9 Hz, 2H), 3.17 (d, *J* = 5.5 Hz, 3H, MeOH), 3.17–3.14 (br, 2H), 3.06–2.90 (br, 2H), 2.16 (t, J = 11.9 Hz, 2H)
^{13}C NMR (125 MHz, DMSO-d_6): δ 168.6, 160.4, 156.3, 152.3, 151.6, 150.7, 135.9, 130.5, 129.2, 128.9, 125.8, 125.0, 124.2, 120.6 (t, $J_{C–F}$ = 214.1 Hz), 119.8, 116.3, 113.6, 110.5, 108.5, 102.4, 81.7, 64.6 (dd, $J_{C–F}$ = 31.2, 10.8 Hz), 48.7, 29.7 (t, $J_{C–F}$ = 25.2 Hz)
^{19}F NMR (471 MHz, CD_3OD): δ −97.9 (d, $J_{F–F}$ = 242.1 Hz, 1F), −105.5 (dtt, $J_{F–F}$, $J_{F–H}$, $J_{F–H}$ = 242.1, 33.0, 11.0 Hz, 1F)
HRMS (ESI+): m/z calcd for $[C_{25}H_{20}F_2NO_5+H]^+$: 452.1304, found 452.1323
Anal. Calcd for $C_{26}H_{23}F_2NO_6$: C, 64.59; H, 4.80; N, 2.90. Found: C, 63.72; H 4.74; N, 2.99.

2. X-ray absorption near edge structure (XANES) spectroscopy

Stock solutions were made with RhoNox-5 dissolved in DMF (20 mM), ferric chloride dissolved in water (10 mM), and ferrous ammonium sulfate hexahydrate (FAS) in water (10 mM). To measure XANES spectra, the stock solutions of RhoNox-5 (20 µL or DMF), ferric chloride (40 µL) or FAS (40 µM), and water (40 µL) were mixed to prepare 4 mM solution of RhoNox-5 and Fe (total 100 µL, 20%DMF in water). XANES experiments were conducted at the beamline BL-9C of Photon Factory in High Energy Accelerator Research Organization (Tsukuba, Japan). The incident X-ray was monochromatized by a Si(111) double-crystal monochromator. XANES spectra were measured in fluorescence mode using a Lytle detector. Sample solutions were measured in microtubes made of a perfluoroalkoxy alkane.

3. Cell culture

HT1080 cells were cultured in Dulbecco's minimum essential medium (Wako) containing 10% fetal bovine

serum (FBS, SERANA), 1% Antibiotic-Antimyocotic (Gibco), and 2 mM glutamine at 37 °C in a 5% CO_2/ 95% air incubator.
For cell viability assay, two days before use, the cells (1.0×10^4 cells/mL) were seeded on a 96-well plate.
For confocal fluorescence imaging experiments, two days before use, the cells (2.0×10^4 cells/mL) were seeded on glass-bottomed dishes (Advanced TC, CELLview Cell Culture Dish, Greiner).

HepG2 cells were cultured in E-MEM (Wako) containing 10% fetal bovine serum (FBS, SERANA), 1% Antibiotic-Antimyocotic (Gibco), and 2 mM glutamine at 37 °C in a 5% CO_2/ 95% air incubator.
For cell viability assay, two days before use, the cells (4.0×10^4 cells/mL) were seeded on a 96-well plate.

A549 cells were cultured in E-MEM (Wako) containing 10% fetal bovine serum (FBS, SERANA), 1% Antibiotic-Antimyocotic (Gibco), and 2 mM glutamine at 37 °C in a 5% CO_2/ 95% air incubator.
For cell viability assay, two days before use, the cells (2.0×10^4 cells/mL) were seeded on a 96-well plate.

PC12 cells were cultured in RPMI-1640 (Wako) containing 10% fetal bovine serum (FBS, SERANA), 1% Antibiotic-Antimyocotic (Gibco), and 2 mM glutamine at 37 °C in a 5% CO_2/ 95% air incubator.
For cell viability assay, two days before use, the cells (4.0×10^4 cells/mL) were seeded on a Collagen TypeI coated Multiwell Plate 96well (Wako).

SH-SY5Y cells were cultured in Dulbecco's minimum essential medium (Wako) containing 10% fetal bovine serum (FBS, SERANA), 1% Antibiotic-Antimyocotic (Gibco), and 2 mM glutamine at 37 °C in a 5% CO_2/ 95% air incubator.
For cell viability assay, one days before use, the cells (4.0×10^4 cells/mL) were seeded on a 96-well plate.

3. Cell viability assay

For checking the inhibitory effect of SiRhoNox-1 on erastin-induced ferroptosis, the cells were treated with erastin (0.1, 0.2, 0.4, 0.8, 1.6, 3.2, 6.4, 12.8 µM, from 1.0 mM stock solution in DMSO) in the presence or absence of deferoxamine mesylate (DFO, 100 µM, from 10 mM stock solution in water) or SiRhoNox-1 (1.0 or 10 µM, from 1.0 mM stock solution in DMSO) in D-MEM (+10% FBS) at 37 °C for 12 h. The cells were treated with Cell Counting Kit-8 (CCK8, DOJINDO) according to the manufacturer's protocol. After incubation for 4 h at 37 °C, absorption at 450 nm was measured by a microplate reader (SpectraMax iD3, Molecular Devices). The cell viabilities were calculated from the absorption.
For evaluating the inhibitory effect of *N*-oxide probes on erastin-induced ferroptosis, the cells were treated with erastin (10 µM, from 1.0 mM stock solution) and the N-oxide probes (0.8, 3.2, 12.8 µM, from DMSO stock solution) in D-MEM (+10% FBS) at 37 °C for 12 h. The cells were treated with Cell Counting Kit-8 (CCK8, DOJINDO) according to the manufacturer's protocol. After incubation for 4 h at 37°C, absorption at 450 nm was measured by a microplate reader (SpectraMax iD3, Molecular Devices). The cell viabilities were calculated from the absorption.
For evaluation of a lethal concentration of RSL3, the cells were treated with RSL3 (8-point, 2.5-fold dilution

from10 μM) in D-MEM (+10% FBS) at 37 °C for 12 h. The cells were treated with Cell Counting Kit-8 (CCK8, DOJINDO) according to the manufacturer's protocol. After incubation for 4 h at 37 °C, absorption at 450 nm was measured by a microplate reader (SpectraMax iD3, Molecular Devices). The cell viabilities were calculated from the absorption.

For the inhibition of RSL3-induced ferroptosis by N-oxide probes, the cells were treated with RSL3 (10 μM, from 1.0 mM stock solution) and the N-oxide probes (0.8, 3.2, 12.8 μM, from DMSO stock solution) in D-MEM (+10% FBS) at 37 °C for 12 h. The cells were treated with Cell Counting Kit-8 (CCK8, DOJINDO) according to the manufacturer's protocol. After incubation for 4 h at 37 °C, absorption at 450 nm was measured by a microplate reader (SpectraMax iD3, Molecular Devices). The cell viabilities were calculated from the absorption.

For analysis of the inhibitory effect of Boc-piperazine-Rhodamine, Si-Rhodamine, Lyso-Rhodamine, the cells were treated with erastin (10 μM, from 1.0 mM stock solution) or RSL3 (10 μM, from 1.0 mM stock solution) and the N-oxide probes (8-point, 2-fold dilution from 25.6 μM) in D-MEM (+10% FBS) at 37 °C for 12 h. The cells were treated with Cell Counting Kit-8 (CCK8, DOJINDO) according to the manufacturer's protocol. After incubation for 4 h at 37 °C, absorption at 450 nm was measured by a microplate reader (SpectraMax iD3, Molecular Devices). The cell viabilities were calculated from the absorption.

For evaluation of cell viability of RhoNox-1, RhoNox-4, SiRhoNox-1, Lyso-RhoNox, the cells were treated with the N-oxide probes (7-points, 2-fold dilution from 32 μM) in D-MEM (+10% FBS) at 37 °C for 12 h. The cells were treated with Cell Counting Kit-8 (CCK8, DOJINDO) according to the manufacturer's protocol. After incubation for 4 h at 37 °C, absorption at 450 nm was measured by a microplate reader (SpectraMax iD3, Molecular Devices). The cell viabilities were calculated from the absorption.

For evaluation of a lethal concentration of FINO2, the cells were treated with FINO2 (8-point, 2-fold dilution from 80 μM) in D-MEM (+10% FBS) at 37°C for 12 h. The cells were treated with Cell Counting Kit-8 (CCK8, DOJINDO) according to the manufacturer's protocol. After incubation for 4 h at 37°C, absorption at 450 nm was measured by a microplate reader (SpectraMax iD3, Molecular Devices). The cell viabilities were calculated from the absorption.

For the inhibition of FINO2-induced ferroptosis by *N*-oxide probes, the cells were treated with FINO2 (10 μM, from 1.0 mM stock solution) and the N-oxide probes (0.8, 3.2, 12.8 μM, from DMSO stock solution) in D-MEM (+10% FBS) at 37 °C for 12 h. The cells were treated with Cell Counting Kit-8 (CCK8, DOJINDO) according to the manufacturer's protocol. After incubation for 4 h at 37 °C, absorption at 450 nm was measured by a microplate reader (SpectraMax iD3, Molecular Devices). The cell viabilities were calculated from the absorption.

For evaluation of a lethal concentration of FIN56, the cells were treated with FIN56 (6-point, 2-fold dilution from 20 μM) in D-MEM (+10% FBS) at 37 °C for 12 h. The cells were treated with Cell Counting Kit-8 (CCK8, DOJINDO) according to the manufacturer's protocol. After incubation for 4 h at 37 °C, absorption at 450 nm was measured by a microplate reader (SpectraMax iD3, Molecular Devices). The cell viabilities were calculated from the absorption.

For the inhibition of FIN56-induced ferroptosis by *N*-oxide probes, the cells were treated with FIN56 (20 μM, from 4.0 mM stock solution) and the N-oxide probes (0, 1.6, 25 μM, from DMSO stock solution) in D-

MEM (+10% FBS) at 37 °C for 12 h. The cells were treated with Cell Counting Kit-8 (CCK8, DOJINDO) according to the manufacturer's protocol. After incubation for 4 h at 37 °C, absorption at 450 nm was measured by a microplate reader (SpectraMax iD3, Molecular Devices). The cell viabilities were calculated from the absorption.

4. Confocal fluorescence microscopy experiments

Confocal fluorescence images were acquired by using an Olympus IX83 microscope equipped with a laser diode illuminator (LDI with 7 laser lines, 89 North), an EMCCD camera (Hamamatsu Photonics, ImagEM), and a disk scan confocal unit (DSU). Fluorescence images were obtained with a FITC filter set (excitation = 470 nm, emission = 516–556 nm, and dichroic mirror = 495 nm). Experiments were performed with a 20× objective lens. For all imaging experiments, Hank′s Balanced Salt Solution (HBSS, Gibco) containing calcium and magnesium without phenol red was used.

For induction of ferroptosis by erastin (Cayman Chemical Company), HT1080 cells were treated with erastin (10 µM, from 1.0 mM stock solution in DMSO), Deferoxamine mesylate (DFO) (100 µM, from 10 mM stock solution in water), SiRhoNox-1 (5.0 µM, from 1.0 mM stock solution in DMSO) in D-MEM with FBS at 37 °C. After incubation for 6.0 h at 37°C, the cells were treated with C11-BODIPY (1.0 µM, from 1 mM stock solution in DMSO, Thermo Fisher Scientific) in HBSS. After incubation for 1.0 h at 37 °C, the cells were washed with HBSS (×1) and then imaged.

For induction of ferroptosis by RSL3 (Cayman Chemical Company), HT1080 cells were treated with RSL3 (500 nM, from 50 µM stock solution in DMSO), Deferoxamine mesylate (DFO) (100 µM, from 10 mM stock solution in water), SiRhoNox-1 (5.0 µM, from 1.0 mM stock solution in DMSO) in D-MEM with FBS at 37 °C. After incubation for 2.0 h at 37 °C, the cells were treated with C11-BODIPY (1.0 μM, from 1 mM stock solution in DMSO) in HBSS. After incubation for 30 min at 37 °C, the cells were washed with HBSS (×1) and then imaged.

For measurement of intracellular ferrous ions in erastin-induced ferroptosis, HT1080 cells were treated with erastin (10 µM, from 1.0 mM stock solution in DMSO), SiRhoNox-1 (5.0, 10 µM, from 1.0 mM stock solution in DMSO) in D-MEM with FBS at 37°C. After incubation for 6.0 h at 37 °C, the cells were treated with RhoNox-4 (1.0 μM, from 1 mM stock solution in DMSO) in HBSS. After incubation for 30 min at 37 °C, the cells were washed with HBSS (×1) and then imaged.

5. Statistics

Error bars indicate standard error of the mean (s.e.m.) unless otherwise indicated. When means of three or more samples were calculated and compared, statistical significance was determined by Student's *t*-test, one-way ANOVA tests with Tukey's test or Dunnett's test.

References in supplementary information

1. T. Hirayama, K. Okuda and H. Nagasawa, *Chem. Sci.*, 2013, **4**, 1250–1256.
2. M. Niwa, T. Hirayama, K. Okuda and H. Nagasawa, *Org. Biomol. Chem.*, 2014, **12**, 6590–6597.
3. T. Hirayama, M. Niwa, S. Hirosawa and H. Nagasawa, *ACS Sens.*, 2020, **5**, 2950–2958.
4. T. Hirayama, H. Tsuboi, M. Niwa, A. Miki, S. Kadota, Y. Ikeshita, K. Okuda and H. Nagasawa, *Chem. Sci.*, 2017, **8**, 4858–4866.
5. M. Niwa, T. Hirayama, I. Oomoto, D. O. Wang and H. Nagasawa, *ACS Chem. Biol.*, 2018, **13**, 1853–1861.
6. T. Hirayama, S. Kadota, M. Niwa and H. Nagasawa, *Metallomics*, 2018, **10**, 794–801.
7. K. Kawai, T. Hirayama, H. Imai, T. Murakami, M. Inden, I. Hozumi and H. Nagasawa, *J. Am. Chem. Soc.*, 2022, **144**, 3793–3803.
